# Harmonizing neuropsychological test data across prospective studies

**DOI:** 10.1002/alz.71186

**Published:** 2026-02-14

**Authors:** Rosita Shishegar, James D. Doecke, Yen Ying Lim, Pierrick Bourgeat, Vincent Dore, Bhargav Tallapragada, Simon M. Laws, Tenielle Porter, Samantha Burnham, Azadeh Feizpour, Ashley Gillman, Michael Weiner, Jason Hassenstab, Christopher C. Rowe, Victor L. Villemagne, Colin L. Masters, Jurgen Fripp, Hamid Sohrabi, Paul Maruff, for the Alzheimer's Disease Neuroimaging Initiative, OASIS‐3, and the Australian Imaging Biomarkers and Lifestyle flagship study of ageing

**Affiliations:** ^1^ The Australian e‐Health Research Centre CSIRO Melbourne Victoria Australia; ^2^ Department of Electrical and Computer Systems Eng Monash University Clayton Victoria Australia; ^3^ The Australian e‐Health Research Centre CSIRO Brisbane Queensland Australia; ^4^ Turner Institute for Brain and Mental Health School of Psychological Sciences Monash University Clayton Victoria Australia; ^5^ Centre for Healthy Ageing Health Futures Institute Murdoch University Murdoch Western Australia Australia; ^6^ School of Psychology Murdoch University Murdoch Western Australia Australia; ^7^ Centre for Precision Health Edith Cowan University Joondalup Western Australia Australia; ^8^ Avid Eli Lilly and Company Indianapolis Indiana USA; ^9^ Florey Institute of Neuroscience and Mental Health The University of Melbourne Parkville Victoria Australia; ^10^ Department of Molecular Imaging & Therapy Austin Health Heidelberg Victoria Australia; ^11^ Center for Imaging of Neurodegenerative Diseases University of California San Francisco San Francisco California USA; ^12^ Institute of Clinical and Translational Sciences Washington University School of Medicine St. Louis Missouri USA; ^13^ Department of Psychiatry University of Pittsburgh Pittsburgh Pennsylvania USA; ^14^ Cogstate Ltd Melbourne Victoria Australia

**Keywords:** clinical–pathological groups, data harmonization, imputation, longitudinal studies, machine learning, neuropsychological tests

## Abstract

**INTRODUCTION:**

Alzheimer's disease (AD) research relies on large datasets and advanced statistical models. However, individual population studies often lack sufficient sample size for conclusive results. Harmonizing cognitive test data across studies can address this gap, despite differences in testing protocols. This study harmonizes cognitive data from three major AD cohorts to support robust clinical–pathological modelling.

**METHODS:**

Information from the Alzheimer's Disease Neuroimaging Initiative (*N* = 1446); Australian Imaging, Biomarkers and Lifestyle (*N* = 1764); and Open Access Series of Imaging Studies‐3 (*N* = 440) were integrated, including cognitive scores, demographics, genetics, and clinical and neuroimaging data. Neuropsychological tests relevant to AD were harmonized using MissForest, a machine learning–based imputation method. Validation involved assessing imputation accuracy and analyzing composite cognitive scores across clinical–pathological groups.

**RESULTS:**

Imputation showed high accuracy (mean absolute error ≤ test–retest variability in cognitively unimpaired participants). Composite scores reflected known disease patterns with significant stratification across clinical–pathological groups.

**DISCUSSION:**

The validated harmonization approach demonstrated reliable imputation, enabling more powerful AD models and supporting future diagnostic and therapeutic advances.

## Highlights

- MissForest imputation reliably harmonized cognitive test scores across Alzheimer's disease (AD) studies.
- Harmonized dataset included 1446 Alzheimer's Disease Neuroimaging Initiative; 1764 Australian Imaging, Biomarkers and Lifestyle; and 440 Open Access Series of Imaging Studies‐3 subjects.
- Mean absolute errors (MAEs) ≤ test–retest variability in cognitively unimpaired participants for most tests.
- Digit Symbol Coding and Trail‐Making Test Part B MAEs slightly > test—retest, < individual variation.
- Composite scores had lower baseline and faster decline with increasing AD severity.

## 1 BACKGROUND

Understanding clinical–pathological relationships along the Alzheimer's disease (AD) continuum has recently accelerated, largely owing to: (1) imaging and fluid biomarkers of amyloid beta (Aβ) and tau; (2) long‐running natural history cohorts that prospectively follow individuals at elevated risk; and (3) sophisticated statistical models that integrate baseline risk factors such as age and apolipoprotein E (*APOE*) ε4 carriage with longitudinal change in Aβ load, hippocampal volume, and cognition.^1^ These multimodal models have underpinned early‐detection algorithms, prognostic tools, and the design of disease‐modifying therapy trials.^2^, ^3^

Developing robust clinical–pathological models of AD requires large datasets.^4^ Although most observational cohorts enroll hundreds of participants, sample size collapses when complex interactions or predictive hypotheses are examined, reducing statistical power and limiting model relationships between changes in biological markers and downstream clinical outcomes, particularly for interactions among cognition, multiple biomarkers, demographic characteristics, and genetic risk factors.^5^ Replicating results across separate cohorts or performing meta‐analyses increases confidence^6^ but treats each study as an indivisible unit, preventing joint analysis of subtle effects. A practical alternative is merging participant‐level data from multiple cohorts into a single harmonized dataset.^7^

Demographic characteristics including age, sex, and *APOE* ε4 carriage align easily across cohorts, yet measures of Aβ load, brain volumes, or cognition are usually acquired with cohort‐specific protocols, requiring mathematical transformation before combination.^8^ Although approaches for integration of Aβ and brain volume data have matured,^9^, ^10^, ^11^ methods for integrating cognitive data are still developing.^12^, ^13^

All AD natural history cohorts assess episodic memory, attention, executive function, and language as changes in these domains are central clinical manifestations. However, the precise tests used, their outcome measures, and scoring conventions across studies, and some domains may be absent. Differences in cognitive test protocols between studies falls into four patterns:^13^ (1) different tests of the same cognitive function (e.g., Rey Auditory Verbal Learning Test [RAVLT],^14^ California Verbal Learning Test Second Edition [CVLT–II],^15^ Selective Reminding Test Free Recall [SRT‐FREE]^16^, ^17^ for episodic memory); (2) identical tests summarized with different indices (speed vs. accuracy); (3) conceptually related tasks such as Digit Symbol Coding, Trail Making Test (TMT), or conflicting color–word tests; and (4) divergent scoring manuals (e.g., clock drawing^18^). These discrepancies create non‐random missingness and cohort‐specific errors, posing obstacles when building domain composites or trial outcomes such as Preclinical Alzheimer Cognitive Composite (PACC).^19^

Integrating cognitive data across studies must address differing domains and test variables, distributions, non‐random missing data, and study‐specific variation,^12^, ^13^ challenges accentuated when aggregating outcomes that combine multiple tests, including domain‐specific composites, or clinical trial outcomes (e.g., PACC).^19^

**RESEARCH IN CONTEXT**

1. **Systematic review**: Cross‐study harmonization of Alzheimer's disease (AD) cognitive data is challenged by test variability. Despite its importance for clinical–pathological modelling, machine learning (ML)‐based harmonization and multi‐cohort validation remain limited. We previously showed MissForest accurately imputes and harmonizes verbal memory tests in Australian Imaging, Biomarkers and Lifestyle (AIBL) and Alzheimer's Disease Neuroimaging Initiative (ADNI). Here, we extend to three independent datasets, validating individual cognitive scores and assessing composite trajectories across clinical–pathological groups over time.
2. **Interpretation**: Neuropsychological data from AIBL, ADNI, and Open Access Series of Imaging Studies‐3 natural history cohorts were harmonized via ML imputation. We rigorously evaluated accuracy, precision, and the validity of composite scores derived from the harmonized data. Errors for individual and composite scores were below clinically meaningful thresholds. Furthermore, composite scores from harmonized data were highly sensitive to established AD progression models.
3. **Future directions**: The harmonized cognitive dataset enables integration with demographic, biological, and genetic data from the same individuals to support development and evaluation of advanced clinical–pathological models of AD.

Integration of cognitive data requires harmonization, defined as transforming related outcomes to equivalent response or scale.^20^, ^21^ Three approaches dominate:^22^ (1) Simple standardization (linear or *z* scaling) allows pooling of analogous variables^20^, ^23^, ^24^ but ignores psychometric differences. (2) Latent‐variable models rely on “anchor” tests to estimate a common factor structure.^25^ While standardization methods require complete data or listwise deletion, and latent‐variable methods can handle missing item responses within administered tests using full information maximum likelihood, neither accommodates structural missingness by design, in which entire tests are systematically absent from specific cohorts due to different data collection protocols. (3) Imputing unadministered tests with multiple imputation or maximum‐likelihood methods^26^, ^27^, ^28^ is more flexible but often oversmooths the data.

Recently we introduced a machine learning (ML)‐based harmonization approach using MissForest,^29^ an iterative imputation method based on random forest (RF).^30^ Capturing complex, non‐linear interactions, MissForest accommodates mixed data types and distributional irregularities typical of neuropsychological scores.^31^ Previously, we demonstrated that MissForest accurately imputed verbal memory scores across the Australian Imaging, Biomarkers and Lifestyle (AIBL), and Alzheimer's Disease Neuroimaging Initiative (ADNI) studies, with imputed data showing clinical discrimination equivalent to measured data.^12^ This study extends this methodology to harmonize cognitive data from three major AD cohorts and validates the performance of individual cognitive scores and composite trajectories across different clinical–pathological groups.

## 2 METHODS

### 2.1 Participants

This study was conducted using data drawn from three large international studies, the AIBL,^16^, ^32^ ADNI,^33^, ^34^ and the Knight Alzheimer's Disease Research Center Open Access Series of Imaging Studies‐3 (OASIS).^35^ The procedures developed and applied here are part of an initiative to construct a harmonized cognitive dataset for these three cohorts; namely, the Alzheimer's Dementia Onset and Progression in International Cohorts (ADOPIC) study.^36^ In this study we refer to AIBL, ADNI, and OASIS as parent datasets.

Each cohort included in this study had extensive batteries of neuropsychological tests, selected based on their established sensitivity to cognitive impairment and decline in early symptomatic AD.^16^, ^32^, ^33^, ^34^ However, the specific tests included in the different studies varied. Each parent dataset also included information on demographic characteristics, *APOE* ε4 carriage, and clinical disease stage, as measured using the Clinical Dementia Rating (CDR) global score. For each participant, at each timepoint in each study, a classification of clinical disease stage was available according to the procedures specific to each study.

### 2.2 Inclusion criteria

In addition to the inclusion and exclusion criteria for each study from which the parent datasets were taken, participant inclusion for this study required complete neuropsychological assessments for a minimum of three assessments. This criterion applied in cases in which records contained only demographic information with no neuropsychological test scores, or when records included demographic data along with only the CDR Sum of Boxes and CDR global scores. Following the application of these criteria, one participant with only a single time‐point was excluded from each of the ADNI and AIBL datasets. Additionally, 1109 and 733 individual time‐points were excluded from the ADNI and AIBL cohorts, respectively, although the corresponding participants remained in the study based on their remaining longitudinal data. Furthermore, participants who were diagnosed with non‐AD dementia‐related clinical conditions (e.g., Parkinson disease, vascular disease, frontotemporal dementia) or did not have a recorded diagnosis were excluded. This resulted in a total of 5683 participants with ≥ 3 assessments over an average of 3.75 years (23,131 timepoints in total). For the second part of our analyses, we only include participants with baseline positron emission tomography (PET) data and evidence of AD‐related pathology, excluding those with cognitive symptoms but without Aβ positivity. This resulted in total of 3658 participants with an average of 3 years of follow‐up. The details of the individual and aggregated sample are summarized in Table 1.

**TABLE 1:** Data demographics: demographic and clinical characteristics of the AIBL, ADNI, and OASIS datasets for the merged dataset with baseline Centiloid measures.

	AIBL	ADNI	OASIS	Merged
*N*	1764	1446	448	3658
Sex, *N* female (%)	932 (52.8)	681 (47.1)	232 (51.8)	1845 (50.4)
*APOE* ε4, *N* (%)	700 (39.68)	619 (42.81)	173 (38.62)	1492 (40.79)
Age, years	72.34 ± 6.98 (41.58, 95.41)	74.04 ± 7.32 (55.5, 95.14)	73.54 ± 6.74 (46.03, 91.18)	73.16 ± 7.13 (41.58, 95.41)
Education, years	12.88 ± 3.14 (5,22)	16.23 ± 2.63 (6,20)	15.74 ± 2.64 (8,22)	14.7 ± 3.29 (5,22)
Centiloid	37.85 ± 43.33 (−26.03,167.02)	43.98 ± 45.99 (−42.37,193.48)	22.04 ± 33.05 (−17.16,128.99)	38.33 ± 43.81 (−42.37,193.48)
Follow‐up years	3.39 ± 3.18 (0, 14.49)	3.15 ± 3.26 (0, 13.11)	2.92 ± 2.54 (0, 8.35)	3.22 ± 3.15 (0, 14.49)
Repeated assessments, *N*	2 (1,9)	3 (1,14)	3 (1,12)	3 (1,14)
**Subset with AD related clinical‐pathological groups**				
N Aβ– CDR = 0	639	358	201	1198
N Aβ+ CDR = 0	254	181	89	524
N Aβ+ CDR = 0.5	472	439	84	995
N Aβ+ CDR = 1	174	166	52	433

*Note*: Continuous variables are indicated as follows: mean ± SD (minimum, maximum).

Abbreviations: Aβ, amyloid beta; AD, Alzheimer's disease; ADNI, Alzheimer's Disease Neuroimaging Initiative; AIBL, Australian Imaging, Biomarkers and Lifestyle; *APOE*, apolipoprotein E; CDR, Clinical Dementia Rating; OASIS, Open Access Series of Imaging Studies.

### 2.3 PET

The PET datasets for Aβ were processed using specialized pre‐processing systems designed for each cohort to quantify amyloid accumulation on the Centiloid (CL) scale.^11^ All Aβ PET images were spatially normalized with CapAIBL.^37^ The images were normalized in standardized uptake value ratio images using the whole cerebellum as reference region, before being quantified into CL using the non‐negative matrix factorization approach,^38^ which was previously proposed to harmonize CL quantification across the ADOPIC studies. The Aβ positivity was defined as PET CL value > 25 CL.^11^

### 2.4 Harmonization procedure stage 1: selection of neuropsychological and clinical tests

Detailed cognitive assessments within each ADOPIC cohort facilitated the initial stage of harmonization, which involved identifying the optimal set of tests for harmonization between cohorts. This was achieved by establishment of an expert committee of neuropsychologists, psychology scientists, and statisticians with experience in AD research. This committee developed a set of theoretical guidelines to guide test selection across cohorts. These included: (1) the aspect of cognition assessed was with a performance‐based neuropsychological test (i.e., not subjective rating, or clinical rating scale); (2) the main performance measure for the neuropsychological test had been validated and had established psychometric characteristics; (3) the neuropsychological test could be classified as measuring one main cognitive domain in the context of AD from the domains of episodic memory, executive function, and language (these cognitive domains were selected as they have been shown to be important in clinical–pathological models of early and symptomatic AD); (4) that the test be scored in the method described by the standard instructions and that raw performance data be available for the main outcome measure; (5) that there were tests sufficient to provide an estimate of the PACC (a composite score that is important to be used in clinical trials as well as diagnostic and screening studies), episodic memory, executive function, and language composites; (6) that with the exception of the PACC, no individual test was used as part of more than one cognitive domain; and (7) the Mini‐Mental State Examination (MMSE) was exempted from criteria 1 through 3 to allow its use for computation of the PACC scores from the different datasets. A common naming convention was developed for the main performance measure from each neuropsychological test (the tests selected for evaluation from the parent datasets are listed in Table 2 and the relationship between the consistency of their application in each parent dataset is highlighted with white and gray cells). Note that some measures (i.e., Rey Complex Figure Test, Stroop Test, FAS Fluency) were excluded due to significant inconsistencies in administration and scoring, which compromised data quality. The neuropsychology team prioritized psychometric integrity when selecting measures for this study. Although this approach reduced the dataset size and limited coverage of certain cognitive domains, it ensured reliability and validity for data harmonization.

**TABLE 2:** Missing data in neuropsychological test scores: sample sizes and rates of missing data (%) for each neuropsychological test score included in ADOPIC merged dataset and in the AIBL, ADNI, and OASIS parent datasets. The gray cells indicate 100% missing data.

Neuropsychological test	AIBL (*N* = 8729)		ADNI (*N* = 11,435)		OASIS (*N* = 2967)		Merged (*N* = 23,131)	
	*n*	% missing	*n*	% missing	*n*	% missing	*n*	% missing
MMSE	8720	0.1	11,399	0.3	2965	0.1	23,084	0.2
LMII	8357	4.3	9382	18.0	2958	0.3	20,697	10.5
RAVLT total learning			11,288	1.3			11,288	51.2
RAVLT delayed recall			11,263	1.5			11,263	51.3
RAVLT recognition hits			11,256	1.6			11,256	51.3
CVLT‐II total learning	8223	5.8					8223	64.5
CVLT‐II delayed recall	8165	6.5					8165	64.7
CVLT‐II recognition hits	8023	8.1					8023	65.3
SRT‐FREE					2760	7.0	2760	88.1
TMT‐B			10,918	4.5	2820	5.0	13,738	40.6
Digit symbol coding	8106	7.1					8106	65.0
Digit span forward	8336	4.5	3380	70.4	2966	0.0	14,682	36.5
Digit span backward	8334	4.5	3380	70.4	2961	0.2	14,675	36.6
BNT no cue	8340	4.5	8841	22.7	2958	0.3	20,139	12.9
Category fluency animal total	8164	6.5	11,352	0.7	2960	0.2	22,476	2.8
Verbal fluency switching	8241	5.6					8241	64.4
Average across all tests	8274	5.2	9246	19.1	2918	1.6	12,926	44.1
Breakdown by CDR status								
Average across all tests for CDR = 0	5905	1.0	2504	20.2	2278	29.5	6763	40.6
Average across all tests for CDR = 0.5	1673	3.3	3472	18.6	403	2.7	3522	45.0
Average across all tests for CDR = 1	514	15.5	925	18.7	224	10.6	1043	47.7

*Notes*: N represents the total number of timepoints when all timepoints collapse for a dataset. *n* is the number of available timepoints for each dataset, and the percentage rate of missing is calculated as missing timepoints (*N – n*) divided by N.

Abbreviations: ADNI, Alzheimer's Disease Neuroimaging Initiative; AIBL, Australian Imaging, Biomarkers and Lifestyle; BNT, Boston Naming Test (for AIBL we used the version adapted for Australian English); CDR, Clinical Dementia Rating; CVLT –II, California Verbal Learning Test Second Edition; MMSE, Mini‐Mental State Examination; OASIS, Open Access Series of Imaging Studies; RAVLT, Rey Auditory Verbal Learning Test; SRT‐FREE, Selective Reminding Test Free Recall. TMT‐B, Trail‐Making Test Part B.

### 2.5 Clinical variables

Data for the global score from the CDR scale^39^ were also collected for each participant in each dataset to be used in analyses of the validity of the harmonized cognitive data. CDR has been shown to be the most commonly used and validated measure of dementia diagnosis and severity. For all participants clinical status was defined using the CDR global score of 0, 0.5, ≥ 1.

### 2.6 Harmonization procedure stage 2: creation of merged dataset

The second stage of harmonization of neuropsychological test data across the three parent datasets occurred in a further three steps. First, a standardized system for scoring demographic variables was established; age was expressed in years, and categorical variables were developed for sex (m/f) and *APOE* ε4 carriage (Y/N). Second, a merged dataset was created from the three parent datasets (AIBL, ADNI, OASIS), with each row in this dataset corresponding to a single timepoint for a participant. Each timepoint was treated as an independent observation, regardless of the interval between assessments, which varied across studies (ADNI: visits as frequent as every 6 months; AIBL: 18‐month cycles; OASIS: annual assessments). Each instance of missing data for each neuropsychological test variable was labelled “NA” in the merged dataset. This included cases in which a participant had no data for a given test at a specific timepoint, as well as tests that were not used in that particular parent dataset. For example, all CVLT‐II records from the ADNI and OASIS datasets were marked NA as it was not measured in either study. From this a merged dataset was generated which included the following variables: (1) identifiers: participant ID and dataset ID; (2) demographic measurements: age, sex, years of education, clinical status defined using the CDR global score (CDR = 0, CDR = 0.5, CDR = 1), and a genetic risk factor (carriage of the *APOE* ɛ4 allele); (3) clinical tests: CDR; and (4) tests scores for each neuropsychological test.

### 2.7 Harmonization procedure stage 3: imputation method

For the third step of the harmonization approach, a ML‐based non‐parametric imputation method was applied to impute all missing data in the merged dataset iteratively.^12^ A key feature of this data harmonization procedure is that all missing data are included as targets for replacement by estimated data. Thus, for a specific neuropsychological test (e.g., CVLT), the limited missing data in a parent dataset in which the test was administered (e.g., AIBL dataset) are treated equivalently to data missing entirely in a different cohort in which the test was not included (e.g., OASIS dataset).

The imputation method applied is called MissForest.^29^ It begins with initial estimates (mean scores for continuous values and the median for discrete ones) for the missing values and then iterates over each variable in the merged dataset. The algorithm imputes the missing values of each variable by fitting a RF model, using information from other variables (e.g., other neuropsychological tests and demographic measurements) to predict the missing data in the target variable. The iteration continues until the changes in imputed values between successive iterations fall below a certain threshold or a maximum number of iterations (here *N* = 10) is reached.^29^

The MissForest algorithm explicitly models non‐linear interactions between predictors, including study‐specific characteristics. By including demographics (age, sex, years of education), clinical status (CDR), *APOE* ε4 status, and all available cognitive test data as predictors in the imputation model, the algorithm learns study‐specific patterns and relationships. Importantly, the algorithm treats each timepoint as an independent observation and does not explicitly model time intervals between visits or test–retest effects during imputation. Rather, age and clinical status at each timepoint serve as proxies for disease stage and aging effects, allowing the algorithm to implicitly capture cognitive profiles appropriate to each participant's characteristics at that assessment. This approach allows the harmonization to account for cohort differences in study design and participant characteristics while preserving cohort‐specific data distributions and adapts to the information available at each assessment regardless of visit frequency differences across studies.

The clinical and neuropsychological test scores across the three parent datasets are listed in Table 2. The gray cells highlight the tests not administered for a dataset that were consequently imputed by the ML harmonization approach.

### 2.8 Statistical analyses of data demographics of the parent datasets

Statistical analyses of differences between parent datasets for continuous data (age, years of education, Aβ burden, and length of follow‐up) were assessed using one‐way analysis of variance, while categorical data (sex, *APOE* ε4 status, and proportion of samples in each clinical disease stage subgroup) were examined using *χ* ^2^ testing. A non‐parametric Mann–Whitney *U* test was performed for ordinal data that did not assume a normal distribution.

### 2.9 Validation procedure: determination of the accuracy of harmonized data

The precision of harmonized data was examined in four steps. First, missing data were simulated by withholding a subset of actual data. In this scenario, simulation involved randomly selecting a subset of all the available timepoints in the merged dataset. This approach was designed to simulate at least 30% of the dataset as missing for each test in each repeat (e.g., 30% available MMSE scores from merged dataset). The experiment was subsequently repeated for each cognitive test score. To enhance the reliability of our results and reduce the potential bias from varying subsets of missing data, we repeated this procedure for each test across 50 iterations.

Second, for each simulation, and for each neuropsychological test across the 50 iterations, all missing scores were imputed. Third, the mean absolute error (MAE) was measured as the difference between the imputed and actual values that were withheld before. Then the mean and the standard deviation (SD) of the MAE measures were calculated as a measure of accuracy and precision, respectively. Fourth, the mean and SD of the MAE metric was compared to: (1) the range of each score, (2) the mean and SD of each score across all participants and timepoints, and (3) the test–retest variability of the scores of cognitively unimpaired (CU) Aβ– participants, defined as CDR of 0 and negative Aβ status. We also calculated the percentage of outliers in the predictions for each iteration and each score. An outlier was defined as an imputed score that was > 2.5 SD units from the actual values (with SD estimated from the distribution of actual data).^40^ The percentage of outliers for each score was calculated by dividing the number of outliers by the total number of cases corresponding to the 30% simulated missing data.

In an ideal situation, it is expected that CU Aβ– participants achieve consistent performance scores across successive repeated assessments with zero test–retest variability. We selected CU Aβ– individuals as our reference group because this population should theoretically show the most stable performance over time, providing a conservative benchmark against which to compare error. Any variability in this group is likely to reflect natural test–retest variability rather than cognitive decline, which would be a confounding factor in symptomatic or progressing individuals. One way to characterize the extent to which scores on the test can vary over time within the same individual is to compute a maximum discrepancy score. The maximum discrepancy score consolidates an individual's performance across multiple timepoints on a given neuropsychological test into a single score. It is calculated as the difference between the highest and lowest scores obtained by each participant for each test.^41^ The mean and SD of the maximum discrepancy score for all CU Aβ– participants can then be computed for each neuropsychological test, and these estimates of average variability in the CU Aβ– sample can be used to express the variability associated with repeated assessments for each test.

### 2.10 Validation procedure: validity of harmonized cognitive and clinical scores

To make the harmonized data as generalizable as possible, data from all timepoints for participants who were classified CU, mild cognitive impairment (MCI), and AD in each parent dataset were used in the harmonization process. The accuracy of harmonized data was examined using cognitive composite scores derived from this merged dataset, and such measures will have utility for understanding aspects of cognition across the AD disease spectrum. Furthermore, by using cognitive and clinical scores from all disease stages during the harmonization process, it is possible that the resulting data became overly homogenized (due to the imputation‐based approach) leading to reduced variability and, consequently, diminished sensitivity to AD‐related cognitive impairment and longitudinal cognitive decline. Therefore, to examine the validity and accuracy of composite scores computed from harmonized data, cognitive decline for each composite score over a period of up to 5 years of follow‐up was modelled using linear mixed effects (LME) models applied to the harmonized dataset.

For these analyses a series of composite scores were computed from the final harmonized dataset. Each composite score was generated using the standard method of: (1) computing the mean and SD of the baseline values of the CU group for each neuropsychological test, (2) deriving participant score on each neuropsychological test as a standard score using this group mean and SD, (3) reversing the sign of standard scores for tests for which higher values indicate worse performance (i.e., tests using time or number of errors as outcomes), and (4) computing the average standard score for the tests that contribute to that composite score. Because the AIBL sample contained the greatest number of participants with the most repeated assessments, the tests selected for the computation of composite scores were drawn from this study. Therefore, for the ADNI and OASIS samples harmonized values for the same tests were submitted for imputation analyses. Using this method for each participant a composite score was computed for episodic memory (i.e., logical memory II [LMII] and CVLT‐II [delayed recall]), executive function (i.e., TMT Part B [TMT‐B] and digit symbol coding test), and language (i.e., Boston Naming Test [BNT] no Cue (for AIBL, the version adapted for Australian English was used) and Category Fluency Animal Total). A PACC score (i.e., MMSE, LMII, CVLT‐II delayed recall and digit symbol coding) was also computed because it is used commonly as an outcome in clinical trials.

The LME models included time and its interaction with the clinical disease status, which was categorized into groups based on the CDR global score, and amyloid status, indicated by Aβ positivity or negativity. The group variable represented the clinical–pathological disease status, capturing the progression from normal cognition Aβ negative to potential impairment. Baseline age, sex, years of education, and parent study were included as fixed covariates to account for their effect on cognitive trajectories. Random effects were specified for individuals to model the inter‐participant variability in baseline cognitive performance and rates of cognitive change over time. The random intercepts and slopes for time provide the flexibility to capture the unique progression patterns of cognitive decline or stability in each participant. Importantly, this random effects structure accounts for between‐participant heterogeneity, including potential study‐specific effects arising from differences in cohort design, while testing whether harmonized composites maintain expected clinical–pathological relationships across the disease spectrum.

These models provided two main estimates that allowed measurement of the sensitivity of the composites based on harmonized data. Comparison of the intercepts derived from these models allows inference about the extent to which cognitive impairment is reflected in the clinical–pathological group. For example, it is expected that in individuals with Aβ+ CDR = 0.5, any of the composite scores will be worse than those in Aβ– CDR = 0 or Aβ+ CDR = 0 but not as severely impaired as in Aβ+ CDR = 1 group. Note that the baseline for each participant is an arbitrary time they entered the study, therefore the estimated marginal mean in the context of LME model takes into account the random effects of individuals and models the inter‐participant variability while adjusting for other factors in the model. The second aspect of the LME model to allow comparison is the rate of decline over time that can be detected for each clinical–pathological group. This was modelled using the linear trends of performance over time from each LME model. From these models, the intercept values were calculated as the marginal mean at time zero and the slopes were calculated as estimated marginal trends for each group using the LME models. Standardized effect sizes (Cohen *d*) were compared between the intercept and the slope in the CU group (Aβ– CDR = 0), and each clinical–pathological group. These statistical evaluations were performed using the emmeans (estimated marginal means), and the emtrends (estimated marginal trends) functions from the emmeans R package.^42^ Therefore, the effects sizes use the comparison between estimated mean and differences between estimated slopes while adjusting for other factors in the model. False discovery rate (FDR) adjustment was conducted for multiple comparisons. All statistical analyses were performed in RStudio version 2023.06.1, R version 4.3.1.

## 3 RESULTS

### 3.1 Study sample

#### 3.1.1 Demographic characteristics

Demographic characteristics of the participants in the harmonized dataset and in the three parent datasets are summarized in Table 1. With respect to the cognitive tests, the AIBL parent dataset included the most data points, followed by the ADNI and OASIS parent datasets. Within each parent dataset, most participants were classified as CU at their earliest assessment. Chi‐squared analyses indicated significant differences between the parent datasets for distributions of sex, *APOE* ε4 carriage, and AD‐related clinical–pathological groups (Aβ– CDR = 0, Aβ+ CDR = 0, Aβ+ CDR = 0.5, Aβ+ CDR = 1). These differences reflect the distinct study designs and recruitment strategies of each cohort. Compared to the AIBL parent dataset, there were more females in the OASIS dataset. Examination of participant characteristics with continuous measurement properties indicated parent datasets were different in their mean age, mean years of education, mean level of amyloid, and mean follow‐up years (Table 1). Compared to the AIBL parent dataset, participants in both the ADNI and OASIS datasets had higher years of education. Finally, compared to the AIBL parent dataset, group mean amyloid levels were higher in the ADNI parent dataset and lower in the OASIS parent dataset. The median number of follow‐up assessments was equivalent in the AIBL, ADNI, and OASIS parent datasets, as was the time interval over which repeated assessments occurred (Table 1).

#### 3.1.2 Clinical and neuropsychological test scores

Table 2 presents the number of cases and the percentages of missing data for each outcome measure in each parent dataset and in the merged dataset. As this harmonization approach is based on imputation of missing data, each neuropsychological test for which data were missing completely in a parent dataset (i.e., when the test was not included in the parent study) was defined as having 100% missing data. In Table 2, tests with 100% missing data are indicated by the dark gray shaded blank cells.

Table 2 shows that for the AIBL parent dataset, the average rate of missing data across neuropsychological tests was ≈ 5%, with rates of missing data increased slightly in the CDR = 0.5 and CDR = 1 clinical disease subgroups compared to the CDR = 0 subgroup. Of the individual neuropsychological tests, missing data was most frequent for CVLT‐II recognition and digit symbol coding tests, although for these measures, the rates of missing data remained close to the average computed across all tests. For neuropsychological tests included in the ADNI parent dataset, the average rate of missing data (19.1%) was higher than the AIBL parent dataset. However, there was no difference in rates of missing data for neuropsychological tests between the clinical disease stage subgroups. Of the individual neuropsychological tests, missing data was most frequent for the digit span forward and backward test, for which ≈ 70% of data for these were missing. For the neuropsychological tests in the OASIS parent dataset, the average rate of missing data was very low (1.6%). However, when considered by clinical disease stage, missing data were more frequent in the CDR = 1 subgroup. Of the individual neuropsychological tests, missing data were most frequent for the SRT‐FREE (7%) and for digit symbol coding (5%). When neuropsychological test data were merged across parent datasets, the average rate of missing data was 44.1%, with missing data increasing slightly across the clinical disease stage groups.

### 3.2 Effect of harmonization on distribution of data for neuropsychological test represented in each parent dataset

The data in Table 3 show the estimates of group means and variability estimated from actual data and from data that consisted of actual and imputed data, in which the imputed data had been derived for missing data for the variable from the harmonization procedure (indicated in Table 3 by the “H‐” prefix). The sample size and total number of timepoints for the actual data for each neuropsychological test in each parent dataset are described in Table 2, where it is shown that the sample size of the available data varies between neuropsychological tests.

**TABLE 3:** Group means in the original and harmonized parent datasets after imputation of missing data for the tests used in each of the studies. The gray cells indicate 100% missing data. The light green cells represent the scores in each dataset that have a rate of missing more than 15%.

Neuropsychological test	AIBL (mean ± SD)	H‐AIBL (mean ± SD)	ADNI (mean ± SD)	H‐ADNI (mean ± SD)	OASIS (mean ± SD)	H‐OASIS (mean ± SD)
MMSE	26.76 ± 4.98	26.76 ± 4.98	26.79 ± 3.87	26.78 ± 3.89	27.98 ± 2.93	27.97 ± 2.95
LMII	9.73 ± 5.4	9.38 ± 5.58	8.77 ± 6.2	8.45 ± 6.04	11.29 ± 5.84	11.27 ± 5.85
RAVLT delayed recall	–		4.62 ± 4.58	4.58 ± 4.57	–	
RAVLT total learning	–		35.67 ± 13.83	35.45 ± 13.97	–	
RAVLT recognition hits	–		10.66 ± 4.06	10.58 ± 4.12	–	
CVLT‐II delayed recall	9.78 ± 4.87	9.26 ± 5.17	_		–	
CVLT‐II total learning	46.33 ± 15.9	44.36 ± 17.6	_		–	
CVLT‐II recognition hits	14.42 ± 2.24	13.75 ± 3.31	_		–	
SRT‐FREE	–		_		28.46 ± 8.51	27.12 ± 9.8
TMT‐B	–		116.73 ± 75.2	122.78 ± 79.98	102.93 ± 61.53	109.86 ± 69.17
Digit symbol coding	56.89 ± 17.0	54.77 ± 18.58				
Digit span forward	10.03 ± 2.34	9.93 ± 2.41	6.46 ± 1.16	7.01 ± 1.29	8.32 ± 2.1	8.32 ± 2.1
Digit span backward	6.6 ± 2.19	6.49 ± 2.28	4.52 ± 1.34	5.02 ± 1.31	6.27 ± 2.28	6.27 ± 2.28
BNT no cue	26.97 ± 4.14	26.47 ± 5.0	25.78 ± 5.0	25.91 ± 4.76	26.56 ± 4.11	26.54 ± 4.12
Category fluency animal total	17.83 ± 5.57	17.31 ± 6.0	17.33 ± 6.3	17.27 ± 6.34	18.81 ± 6.23	18.79 ± 6.25
Verbal fluency switching	10.83 ± 3.94	10.44 ± 4.28	–		–	

Abbreviations: ADNI, Alzheimer's Disease Neuroimaging Initiative; AIBL, Australian Imaging, Biomarkers and Lifestyle; BNT, Boston Naming Test (for AIBL we used the version adapted for Australian English); CDR, Clinical Dementia Rating; CVLT –II, California Verbal Learning Test Second Edition; MMSE, Mini‐Mental State Examination; OASIS, Open Access Series of Imaging Studies; RAVLT, Rey Auditory Verbal Learning Test; SD, standard deviation; SRT‐FREE, Selective Reminding Test Free Recall; TMT‐B, Trail‐Making Test Part B.

Table 3 shows that the group means and SDs for each neuropsychological test were consistent between the original datasets and the harmonized dataset, indicating that the harmonization process preserved the original data characteristics.

### 3.3 Validation results: precision of harmonized data for neuropsychological tests with available data

Results in Table 4 show that, except for the verbal fluency switching, estimates of within‐individual variation, the CU group maximum discrepancy scores, were less than the estimate of between‐individual variation (i.e., group SD) for each neuropsychological test. For each test, these two estimates of within‐group variation (Table 4, columns 3 and 4) provide limits against which the variance in the harmonized dataset (MAE for simulated missing data; Table 4, column 5) can be compared. First, the data from the re‐sampling procedure with 30% of the data held out showed that imputed absolute error (MAE) for different samples was very small relative to score range (Table 4, column 2). Also, for most neuropsychological tests, the MAE was less than or equal to the CU maximum discrepancy score. For the digit symbol coding test and the TMT‐B, MAEs were slightly greater than the maximum discrepancy score for each test. However, for both tests, maximum discrepancy score remained less than the estimate of between‐individual variation. The generally high precision for harmonized data was also observed in the analysis of proportion of outlier values from the resampling procedure (Table 4, column 6). This analysis indicated that there were < 1% of outliers in the harmonized data for each neuropsychological test, except the CVLT‐II recognition memory hits and TMT‐B, where they were increased but remained < 3%.

**TABLE 4:** Analyses of precision of harmonization procedure for imputing missing data.

	Actual scores			Imputed scores	
Neuropsychological test scores	Range (Min, Max)	Mean ± SD	Max discrepancy for cognitively unimpaired Aβ– (mean ± SD [range])	MAE for 30% simulated missing (mean ± SD)	Outliers for 30% simulated missing (%)
MMSE*	(0, 30)	26.93 ± 4.25	1.65 ± 1.13 (0, 5)	1.34 ± 0.14	0.17 ± 0.08
LMII*	(0, 25)	9.52 ± 5.9	3.61 ± 2.19 (0, 11)	2.65 ± 0.22	0.02 ± 0.01
RAVLT delayed recall	(0, 15)	4.62 ± 4.58	3.78 ± 3.01 (0, 13)	1.59 ± 0.31	0.34 ± 0.09
RAVLT total learning	(0, 75)	35.67 ± 13.83	9.83 ± 6.32 (0, 38)	5.42 ± 0.9	0.00 ± 0.00
RAVLT recognition hits	(0, 15)	10.66 ± 4.06	2.34 ± 2.32 (0, 12)	2.17 ± 0.46	0.78 ± 0.11
CVLT‐II delayed recall*	(0, 16)	9.78 ± 4.87	2.68 ± 1.63 (0, 9)	1.51 ± 0.37	0.01 ± 0.02
CVLT‐II total learning*	(0, 80)	46.33 ± 15.9	9.28 ± 4.95 (0, 25)	5.57 ± 0.98	0.00 ± 0.01
CVLT‐II recognition hits*	(0, 16)	14.42 ± 2.24	1.33 ± 1.11 (0, 4)	1.31 ± 0.41	3.78 ± 0.69
SRT‐FREE	(0, 48)	28.46 ± 8.51	5 ± 2.8 (0, 10)	4.6 ± 1.12	0.12 ± 0.06
TMT‐B	(5, 300)	113.89 ± 72.82	30.81 ± 27.45 (0, 130)	39.53 ± 7.3	2.08 ± 0.17
Digit symbol coding*	(0, 127)	56.89 ± 17	9.36 ± 5.72 (0, 30)	11.29 ± 2.6	0.20 ± 0.08
Digit span forward*	(0, 16)	8.86 ± 2.54	1.84 ± 1.17 (0, 6)	1.43 ± 0.18	0.08 ± 0.04
Digit span backward*	(0, 14)	6.05 ± 2.22	1.97 ± 1.34 (0, 7)	1.22 ± 0.15	0.22 ± 0.06
BNT no cue*	(0, 30)	26.39 ± 4.57	1.44 ± 1.21 (0, 6)	2.13 ± 0.33	0.69 ± 0.15
Category fluency animal total*	(0, 60)	17.71 ± 6.05	5.33 ± 3.12 (0, 21)	3.37 ± 0.26	0.17 ± 0.04
Verbal fluency switching*	(0, 24)	10.83 ± 3.94	4.03 ± 2.56 (0, 15)	2.46 ± 0.56	0.63 ± 0.14

*Note*: Tests with an asterisk indicate that the tests are administered in the AIBL parent dataset.

Abbreviations: Aβ, amyloid beta; ADNI, Alzheimer's Disease Neuroimaging Initiative; AIBL, Australian Imaging, Biomarkers and Lifestyle; BNT, Boston Naming Test (for AIBL we used the version adapted for Australian English); CDR, Clinical Dementia Rating; CVLT –II, California Verbal Learning Test Second Edition; MAE, mean absolute error; Max, maximum; Min, minimum; MMSE, Mini‐Mental State Examination; OASIS, Open Access Series of Imaging Studies; RAVLT, Rey Auditory Verbal Learning Test; SD, standard deviation; SRT‐FREE, Selective Reminding Test Free Recall; TMT‐B, Trail‐Making Test Part B.

Together these analyses indicated that the data harmonization process had provided reliable estimates of performance for tests with 100% of data missing from a parent dataset, and where missing data was < 100%. As the results of the data harmonization were based on integration of data from all tests, in all participants, and for all timepoints it was necessary to understand the validity of harmonized data by considering the effects of clinical disease status and time on composite scores developed from the harmonized dataset.

### 3.4 Validation results: validity of composite scores derived from harmonized data

**FIGURE 1:**
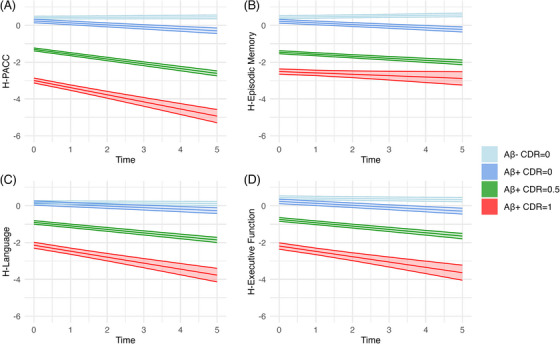
Cognitive trajectories over time stratified by CDR and Aβ status. Longitudinal cognitive decline in harmonized composite scores (A) H‐PACC, (B) H‐episodic memory, (C) H‐language, and (D) H‐executive function. Aβ, amyloid beta; CDR, Clinical Dementia Rating; PACC, Preclinical Alzheimer Cognitive Composite.

**FIGURE 2:**
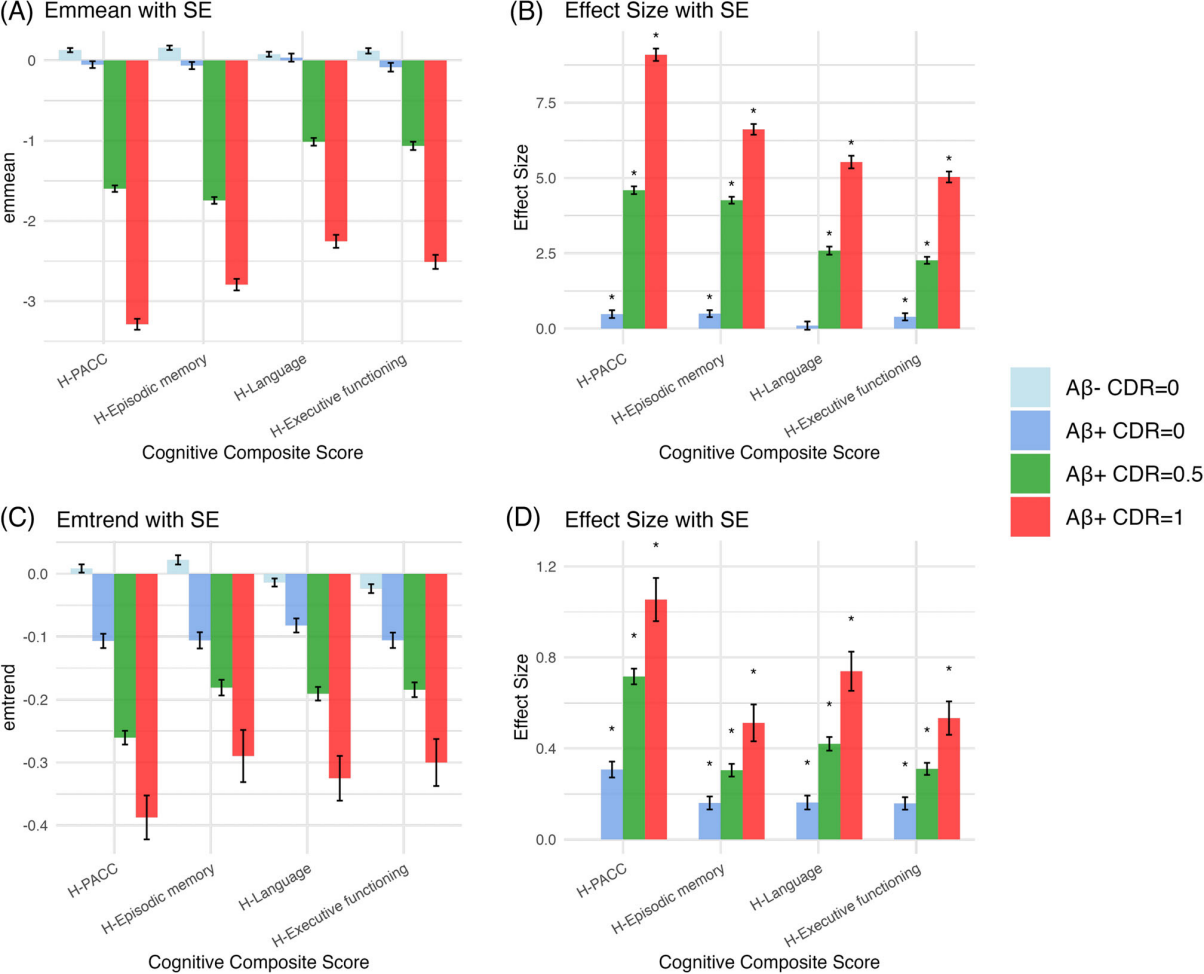
Statistical summary of (A) baseline and (C) rate of decline values for cognitive composite scores, stratified by CDR and Aβ status. Asterisks indicate significant differences (P < 0.001) compared to the reference group, Aβ– CDR = 0; (B) and (D) present the effect sizes of each AD‐related groups compared to the reference group. Aβ, amyloid beta; AD, Alzheimer's disease; CDR, Clinical Dementia Rating; SE, standard error.

For each cognitive composite, the analysis revealed the influence of amyloid status and clinical disease stage on cognitive change over time. A summary of these results is presented in Figure 1, with the statistical comparisons highlighted in Figure 2 and detailed in Tables S1 and S2 in supporting information.

As shown in Figure 1A–D and summarized in the intercept values presented from each model in Figure 2A,B, compared to the Aβ– CDR = 0 group, there was a small but significant difference in the Aβ+ CDR = 0 group for each composite score except for the language. Also, a significantly worse performance was observed in the Aβ+ CDR = 0.5, and Aβ+ CDR = 1 groups for each of the composite scores. Estimated effect sizes reflecting these group differences increase substantially with AD‐related clinical severity increase, with this increase greatest for the PACC and episodic memory composites. In comparison, effect sizes for the language and executive function composites indicated smaller magnitude impairment.

Figures 1 and 2A–D summarize, for each cognitive composite, the differences over time in each clinical disease severity group, with rates of change in each clinical disease severity group compared to that in the Aβ– CDR = 0 group. The results of the analyses show that there was a statistically significant decrease over time for each of the cognitive composite scores in the Aβ+ CDR = 0, Aβ+ CDR = 0.5, and Aβ+ CDR = 1 groups.

## 4 DISCUSSION

This study demonstrates that the MissForest harmonization approach can support reliable and accurate aggregation of cognitive test data, collected prospectively in large natural history cohorts of adults with AD. The resultant increase in sample sizes allows modelling of complex clinical–pathological relationships across the AD clinical disease spectrum. The development of sophisticated and generalizable brain behavior models of AD is improved as the size of the samples from which such models are developed is increased.^43^ Larger sample sizes improve the precision of estimates derived from such models, as well as our understanding of the inter‐relationships between cognition and confounders, both at single timepoints, and over time or in the multivariable space. One approach to increasing sample sizes for developing clinical–pathological models of AD has been to combine data on relevant biological and clinical outcomes from multiple study cohorts.^12^

Combination of the same data from multiple cohorts is relatively straightforward; however, it can be more complex when different prospective studies of AD have used different neuropsychological tests to measure change in cognition. To date, attempts to harmonize neuropsychological test data across multiple cohorts have been conducted through: (1) computing composite measures using the tests from each sample, even if they differ, and then generating composite scores using within sample estimates variation;^44^ (2) computing cognitive domain–specific composite scores, or clinical trial outcome composite scores (i.e., PACC) for each sample, based on performance on the relevant tests from that sample, and then treating these as dependent variables in analyses that integrate data across samples; and (3) computing composite scores within each sample, based on their neuropsychological test battery and then harmonizing the composite scores using item response theory, or using a K‐nearest neighbor (KNN) non‐parametric approach to impute missing data for a small subset of tests that have been used to generate a clinical trial–related composite score (i.e., the PACC). Each of these methods are problematic for harmonized cognitive data, as they assume: (1) sample‐specific estimates of variance reflect the same factors in the different study cohorts; (2) different tests of the same cognitive domain, used in different studies, have equivalent metric characteristics; (3) harmonization of composite scores can limit the effect of sample specific differences in individual test characteristics, or sample‐specific variance on these tests; and (4) that data harmonization based on the KNN values from a small set of cognitive tests will provide a robust estimate of missing data. By defining harmonization as a technique for recalibrating scores from different but related tests onto a unified scale to enable data pooling,^12^, ^20^, ^22^, ^45^, ^46^ we distinguish our approach from traditional psychometric equating; rather than assuming test equivalence, our objective is to model the underlying relationships between all available cognitive measures for robust cross‐study integration, which is particularly crucial when entire tests are missing from specific cohorts. Consequently, the limitations of previous attempts to harmonize data were avoided by: (1) using all cognitive data from the study in the harmonization progress; (2) extending this harmonization dataset to include individuals with each AD clinical disease stage, and also data for their repeated assessments; and (3) using the MissForest algorithm that maintains the original data distribution and variance by using conditional modelling, crucial for harmonizing multiple cohorts unlike KNN's that rely on averaging neighbors, which can lead to oversmoothing, erasing cohort‐specific heterogeneity critical for cross‐study comparisons.

For each test in the merged dataset, missing data could reflect data missing at random, or an absence of data because a test was not administered in a cohort. The MissForest approach treats these two sources of missing data equivalently. However, the effect of the MissForest harmonization on data estimates can be checked by comparing the characteristics of data distributions for individual tests when missing data are allowed and after harmonization and imputation of data for missing cases. Table 3 summarizes these analyses and shows that for each test in each parent dataset, estimates of the distribution mean and SD are almost identical before and after harmonization. Slight increases in group means after harmonization occurred mainly in tests with high proportions of missing data (e.g., ≥ 15% missingness in digit span forward/backward, BNT no cue, and LMII).

Investigation of the outcome of the harmonization were then extended to include distributional characteristics estimated with all sources of missing data included (Table 4). For all tests the estimated MAEs were less than the SD of the sample of actual data, and less than the maximum discrepancy scores for majority of the tests, reflecting the potential for normal variability in test performance. Together, these characteristics indicate that for all variables, harmonization of imputed data has provided estimates with a precision that has not decreased compared to the actual data.

Data from neuropsychological tests applied in prospective natural history studies of AD show that composite scores, constructed by combining outcome measures from theoretically related neuropsychological tests, have greater sensitivity to both clinical disease severity, the effect and level of biomarkers, modulation of biomarker effects by genetic characteristics, and associations with brain structure and volume.^19^, ^47^ While previous approaches to data harmonization have sought to harmonize composite scores,^13^ harmonization of the outcomes from individual tests in the current study allows these individual scores to be constructed into composite scores. Another important aspect of the current harmonization approach is that a term for time was not included in any harmonization procedure. Rather all test scores from all timepoints in all participants were aggregated into a single dataset with a large sample size that provided a solid foundation for harmonization. It is possible that aggregation of the data in this way may reduce the sensitivity of outcomes to AD‐related symptomatic change. Thus, one approach to establishing the validity of the harmonization procedure, applied to the individual tests, is to determine the extent to which composite scores generated from these individual tests are sensitive to AD disease characteristics that have been well demonstrated to influence cognition. To do this, episodic memory, executive function, language, and PACC scores were tested using LME models, with results for each composite score as expected per clinical disease group. The CU Aβ– group showed relatively stable performance over the 5 years of assessment. Composite score values at the *y* intercept for the Aβ+ CDR = 0 group started from a similar point as the CU Aβ– group but declined subtly across the 5 years. Both Aβ+ symptomatic groups had lower intercepts and declined substantially across the 5 years.

Magnitudes of differences between the CU Aβ– group and each of the cognitively impaired groups showed measurable cognitive decline at baseline, and increasing cognitive decline over time. In participants in the CDR = 0.5 group the magnitude of impairment at the *y* intercept was ≈ 1.5 SD below the CU Aβ– group for the PACC and episodic memory composites. In the same group, the magnitude of impairment at baseline was slightly less than ≈ 1 SD for the language and executive function composite. A quantitatively more severe pattern of impairment at baseline was evident from the *y* intercepts in the Aβ+ CDR = 1 group, with impairment in the PACC and episodic memory composites almost 3 SDs below the Aβ– CDR = 0 control group, while the *y* intercept difference between the Aβ– CDR = 0 and CDR = 1 groups was slightly less (< 2 SD) for language and executive function. Together these results recapitulate the patterns of cognitive impairment known to occur in symptomatic AD.^19^, ^48^ By contrast, and as expected, there was little to no impairment at baseline for any cognitive composite in the Aβ+ CDR = 0 group.

It is well known that both preclinical (Aβ+ CDR = 0) and symptomatic AD are characterized by decline in cognition.^5^ The slopes of change over time for each composite were consistent with this disease characteristic. First, decline was greater in the adults with dementia (Aβ+ CDR = 1) than in adults with MCI Aβ+ (CDR = 0.5) and this occurred for each composite score. The preclinical AD group also showed significantly greater decline on each composite score compared to CU Aβ–, and though the magnitude of this was subtle, it was consistent with that observed previously in meta‐analytic estimates of cognitive decline in preclinical AD.^5^ Taken together, the level of impairment on each cognitive composite score observed at baseline and the decline on the same cognitive composite over the 5‐year study period in each clinical group indicates strongly that estimates of test performance arising from the application of the MissForest harmonization procedure retain their sensitivity to AD‐related cognitive decline.

Overall, this study shows that the harmonized cognitive tests and the calculated composite scores developed here using the ML harmonization approach are accurate and precise. This confirms that the ML harmonization method has utility for aggregating data from different neuropsychological tests used in different AD prospective studies. Harmonized neuropsychological test data from the ADOPIC consortium will provide a large dataset for development and refinement of AD clinical–pathological models. Our approach will serve as a platform for future comparisons to other ML or statistical harmonization methods, including item response theory and confirmatory factor analysis.

## CONFLICT OF INTEREST STATEMENT

C.L.M. reports ad hoc consultancy speaking engagements and scientific advice with Actinogen, Acumen, Alterity, Biogen, Eisai, Eli Lilly, and Roche. C.C.R. has received research grants from National Health and Medical Research Council (NHMRC) of Australia, Medical Research Future Fund (MRFF), Enigma Australia, Biogen, Eisai, Roche, and Abbvie. He is on the scientific advisory board for Enigma Biomedical Group and consulted for Prothena, Roche, Eisai, Eisai Australia, Eli Lilly Australia, and Novo Nordisk Australia. M.W. serves on editorial boards for *Alzheimer's & Dementia*, and the *Journal for Prevention of Alzheimer's Disease* (JPAD). He has served on advisory boards for Acumen Pharmaceutical, Alzheon, Inc., Amsterdam UMC, MIRIADE, Cerecin, Merck Sharp & Dohme Corp., NC Registry for Brain Health, ProMIS Neurosciences, Inc., and REGEnLIFE. He also serves on the USC ACTC grant, which receives funding from Eisai. M.W. has provided consulting to Acadia Pharmaceuticals, Boxer Capital, LLC, Cerecin, Inc., Clario, Dementia Society of Japan, Dolby Family Ventures, Eisai, Guidepoint, Health and Wellness Partners, Indiana University, LCN Consulting, MEDA Corp., Merck Sharp & Dohme Corp., NC Registry for Brain Health, NovoNordisk, Owkin France, ProMIS Neurosciences, Prova Education, Sai MedPartners, T3D Therapeutics, University of Southern California (USC), and WebMD. M.W. has acted as a speaker/lecturer for BrightFocus Foundation, China Association for Alzheimer's Disease (CAAD), and Taipei Medical University, as well as a speaker/lecturer with academic travel funding provided by: AD/PD Congress, Amsterdam UMC, Banner Health, Cleveland Clinic, CTAD Congress, Foundation of Learning; Health Society (Japan), Kenes, U. Madison Wisconsin, U. Penn, U. Toulouse, Japan Society for Dementia Research, Korean Dementia Society, Merck Sharp & Dohme Corp., National Center for Geriatrics and Gerontology (NCGG; Japan), University of Madison Wisconsin, University of Southern California (USC). M.W. holds stock options with Alzeca, Alzheon, Inc., ALZpath, Inc., and Anven. S.C.B. is an employee and shareholder of Eli Lilly and Co. Ltd. The other authors have nothing to disclose. Author disclosures are available in the supporting information.

## CONSENT STATEMENT

All AIBL participants gave written consent for publication of de‐identified data. All ADNI participants signed written informed consent for participation in the ADNI, as approved by the institutional review board at each participating center. All OASIS‐3 participants consented to the use of their data by the scientific community and data sharing terms have been approved by the Washington University Human Research Protection Office.

## Supporting information



Supporting Information

Supporting Information

## Data Availability

A software application implementing the proposed harmonization methodology is available upon reasonable request from the corresponding author (rosita.shishegar@csiro.au). We encourage independent researchers to use this implementation and the proposed validation procedure to conduct comparative evaluations with alternative harmonization methods and assess generalizability across datasets.
