# Supplementary material for: Harmonizing neuropsychological test data across prospective studies
Source: Alzheimers Dement. 2026 Feb 14;22(2):e71186. doi: 10.1002/alz.71186 (PMC12906370; doi:10.1002/alz.71186)
Supplement: Supplementary file 1 — Supporting Information [file ALZ-22-e71186-s001.docx]

## Harmonizing Neuropsychological Test Data Across Prospective Studies

Rosita Shishegar^1,2^, James D. Doecke^3^, Yen Ying Lim^4^, Pierrick Bourgeat^3^, Vincent Dore^1^, Bhargav Tallapragada^5,6^, Simon M. Laws^7^, Tenielle Porter^7^, Samantha Burnham^8^, Azadeh Feizpour^9,10^, Ashley Gillman^3^, Michael Weiner^11^, Jason Hassenstab^12^, Christopher C. Rowe^9,10^, Victor L. Villemagne^13^, Colin L. Masters^9^, Jurgen Fripp^3^, Hamid Sohrabi^5,6^, Paul Maruff^14^, for the Alzheimer’s Disease Neuroimaging Initiative^*^, the AIBL Research

1.The Australian e-Health Research Centre, CSIRO, Melbourne, Australia; 2. Department of Electrical and Computer Systems Eng, Monash University, Clayton VIC, 3800, Australia; 3. The Australian e-Health Research Centre, CSIRO, Brisbane, Australia;

4. Turner Institute for Brain and Mental Health, School of Psychological Sciences, Monash University, Melbourne, Australia; 5. Centre for Healthy Ageing, Health Futures Institute, Murdoch University, Murdoch, Western Australia, Australia; 6. School of Psychology, Murdoch University, Murdoch, Western Australia, Australia; 7. Centre for Precision Health, Edith Cowan University, Joondalup, Western Australia, Australia; 8. Avid, Eli Lilly and Company, Indianapolis, IN USA; 9. Florey Institute of Neuroscience and Mental Health, The University of Melbourne, 30 Royal Parade, Parkville, VIC 3052, Australia; 10. Department of Molecular Imaging & Therapy, Austin Health, 145 Studley Road, Heidelberg, VIC 3084, Australia; 11.Center for Imaging of Neurodegenerative Diseases, University of California-San Francisco, San Francisco, CA, USA; 12. Institute of Clinical and Translational Sciences, Washington University School of Medicine, St Louis, USA; 13. Department of Psychiatry, University of Pittsburgh, 3811 O’Hara Street, Pittsburgh, PA 15213, USA; 14. Cogstate Ltd, Melbourne, VIC, Australia;

*Data used in preparation of this article were obtained from the Alzheimer's Disease Neuroimaging Initiative (ADNI) database (adni.loni.usc.edu). As such, the investigators within the ADNi contributed to the design and implementation of ADNI and/or provided data but did not participate in analysis or writing of this report. A complete listing of ADNI investigators can be found at: http://adni.loni.usc.edu/wp-content/uploads/how_to_apply/ADNI_Acknowledgement_List.pdf

Supplementary Material

**Table S1. Statistical summary of the baseline values for cognitive composite scores**

| **Composite score** | **Group** | **emmean** | **SE** | **95% CI** | **p-values** | **Estimate** | **SE** | **95% CI** |
| --- | --- | --- | --- | --- | --- | --- | --- | --- |
| H-PACC | Aβ- CDR=0 | 0.13 | 0.03 | [0.06-0.19] | — | — | — | — |
|  | Aβ+ CDR=0 | -0.05 | 0.04 | [-0.16-0.05] | **0.0002** | 0.48 | 0.13 | [0.23-0.73] |
|  | Aβ+ CDR=0.5 | -1.60 | 0.04 | [-1.70-1.50] | **<.0001** | 4.60 | 0.13 | [4.34-4.85] |
|  | Aβ+ CDR=1 | -3.29 | 0.07 | [-3.46-3.12] | **<.0001** | 9.10 | 0.21 | [8.69-9.50] |
| H-Episodic memory | Aβ- CDR=0 | 0.16 | 0.03 | [0.09-0.23] | — | — | — | — |
|  | Aβ+ CDR=0 | -0.06 | 0.04 | [-0.18-0.05] | **<.0001** | 0.50 | 0.12 | [0.27-0.72] |
|  | Aβ+ CDR=0.5 | -1.75 | 0.04 | [-1.85-1.64] | **<.0001** | 4.26 | 0.11 | [4.04-4.49] |
|  | Aβ+ CDR=1 | -2.79 | 0.07 | [-2.97-2.62] | **<.0001** | 6.62 | 0.18 | [6.27-6.97] |
| H-Language | Aβ- CDR=0 | 0.08 | 0.03 | [-0.00-0.15] | — | — | — | — |
|  | Aβ+ CDR=0 | 0.03 | 0.05 | [-0.09-0.16] | 0.4669 | 0.10 | 0.14 | [-0.17-0.37] |
|  | Aβ+ CDR=0.5 | -1.01 | 0.05 | [-1.13-0.90] | **<.0001** | 2.59 | 0.13 | [2.33-2.85] |
|  | Aβ+ CDR=1 | -2.25 | 0.08 | [-2.46-2.05] | **<.0001** | 5.53 | 0.21 | [5.12-5.94] |
| H-Executive functioning | Aβ- CDR=0 | 0.12 | 0.03 | [0.04-0.20] | — | — | — | — |
|  | Aβ+ CDR=0 | -0.09 | 0.05 | [-0.22-0.05] | **0.0011** | 0.39 | 0.12 | [0.16-0.63] |
|  | Aβ+ CDR=0.5 | -1.07 | 0.05 | [-1.19-0.94] | **<.0001** | 2.27 | 0.12 | [2.04-2.50] |
|  | Aβ+ CDR=1 | -2.51 | 0.09 | [-2.73-2.29] | **<.0001** | 5.04 | 0.18 | [4.68-5.39] |

*emmean = estimated marginal mean (baseline values), SE = Standard Error, CI = Confidence Intervals, Reference group = AB- CDR=0; Statistically significant p-values are shown in bold.*

**Table S2. Statistical summary of the cognitive decline for cognitive composite scores**

| **Composite score** | **Group** | **emtrend** | **SE** | **95% CI** | **p-values** | **Estimate** | **SE** | **95% CI** |
| --- | --- | --- | --- | --- | --- | --- | --- | --- |
| H-PACC | Aβ- CDR=0 | 0.01 | 0.01 | [-0.00-0.02] | — | — | — | — |
|  | Aβ+ CDR=0 | -0.11 | 0.01 | [-0.13-0.08] | **<.0001** | 0.31 | 0.04 | [0.24-0.38] |
|  | Aβ+ CDR=0.5 | -0.26 | 0.01 | [-0.28-0.24] | **<.0001** | 0.72 | 0.03 | [0.65-0.78] |
|  | Aβ+ CDR=1 | -0.39 | 0.03 | [-0.46-0.32] | **<.0001** | 1.05 | 0.09 | [0.87-1.24] |
| H-Episodic memory | Aβ- CDR=0 | 0.03 | 0.01 | [0.01-0.04] | — | — | — | — |
|  | Aβ+ CDR=0 | -0.09 | 0.01 | [-0.11-0.07] | **<.0001** | 0.25 | 0.03 | [0.20-0.31] |
|  | Aβ+ CDR=0.5 | -0.11 | 0.01 | [-0.13-0.09] | **<.0001** | 0.3 | 0.03 | [0.25-0.36] |
|  | Aβ+ CDR=1 | -0.07 | 0.04 | [-0.15-0.00] | **<.0001** | 0.22 | 0.08 | [0.06-0.39] |
| H-Language | Aβ- CDR=0 | -0.01 | 0.01 | [-0.03-0.00] | — | — | — | — |
|  | Aβ+ CDR=0 | -0.08 | 0.01 | [-0.10-0.06] | **<.0001** | 0.16 | 0.03 | [0.10-0.22] |
|  | Aβ+ CDR=0.5 | -0.19 | 0.01 | [-0.21-0.17] | **<.0001** | 0.42 | 0.03 | [0.36-0.48] |
|  | Aβ+ CDR=1 | -0.33 | 0.04 | [-0.40-0.26] | **<.0001** | 0.74 | 0.09 | [0.57-0.91] |
| H-Executive functioning | Aβ- CDR=0 | -0.02 | 0.01 | [-0.04-0.01] | — | — | — | — |
|  | Aβ+ CDR=0 | -0.11 | 0.01 | [-0.13-0.08] | **<.0001** | 0.16 | 0.03 | [0.10-0.22] |
|  | Aβ+ CDR=0.5 | -0.18 | 0.01 | [-0.21-0.16] | **<.0001** | 0.3 | 0.03 | [0.25-0.36] |
|  | Aβ+ CDR=1 | -0.29 | 0.04 | [-0.37-0.21] | **<.0001** | 0.51 | 0.08 | [0.35-0.67] |

*emtrend = estimated marginal trend (slope), SE = Standard Error, CI = Confidence Intervals, Reference group = AB- CDR=0; Statistically significant p-values are shown in bold.*
